# Supplementary material for: Pre-schoolers’ images, intergroup attitudes, and liking of refugee peers in Germany
Source: PLoS One. 2023 Feb 2;18(2):e0280759. doi: 10.1371/journal.pone.0280759 (PMC9894457; doi:10.1371/journal.pone.0280759)
Supplement: S1 File — (DOCX) [file pone.0280759.s001.docx]

**SUPPORTING INFORMATION**

**Pre-schoolers’ images, intergroup attitudes, and liking of refugee peers in Germany.**

Iris Würbel^1,*^, Patricia Kanngiesser^1,2^

^1^Faculty of Education and Psychology, Freie Universität Berlin, Berlin, Germany

^2^School of Psychology, University of Plymouth, Plymouth, UK

*Corresponding author: Iris Würbel, iris.wuerbel@fu-berlin.de

[**Additional information on methods 1**](#_Toc86336062)

[Recruiting sites in Berlin 1](#_Toc86336063)

[Study materials 2](#_Toc86336064)

[**Additional information on data analysis 5**](#_Toc86336065)

[**Additional information on results 7**](#_Toc86336066)

[**References 9**](#_Toc86336067)

# Additional information on methods

| **S1 Table. Percentage of people without German citizenship and with migration history in Berlin overall and in the districts of Berlin where kindergartens in the sample were located.** | | |
| --- | --- | --- |
| district | percentage of people without  German citizenship in … | percentage of people with  migration history in … |
| **Berlin** | **18.2%** | **30.0%** |
| Steglitz-Zehlendorf | 11.9% | 21.1% |
| Tempelhof-Schöneberg | 19.4% | 33.4% |
| Mitte | 33.9% | 48.9% |
| Friedrichshain-Kreuzberg | 25.9% | 38.0% |
| Lichtenberg | 16.7% | 17.9% |
| *Note.* Source: Amt für Statistik Berlin-Brandenburg [1]. Migration history here includes persons who have a migration history themselves and/or persons whose parents live in the same household and have a migration history. | | |

## **Recruiting sites in Berlin**

## **Study materials**

| **S2 Table.**  **Parental questionnaire on parents’ own and their children’s contact experiences with refugees.** | | | | | |
| --- | --- | --- | --- | --- | --- |
| How often …  (German: Wie oft …) | Never (nie) | Rarely (selten) | Occasionally (gelegentlich) | Often (oft) | Very often (sehr oft) |
| … have **you** had contact with refugees until now?  (… hatten **Sie** bisher Kontakt zu Geflüchteten?) |  |  |  |  |  |
| … has **your child** had contact with refugee adults until now?  (…hatte Ihr Kind bisher Kontakt zu geflüchteten Erwachsenen?) |  |  |  |  |  |
| … has **your child** had contact with refugee adults until now?  (…hatte Ihr Kind bisher Kontakt zu geflüchteten Kindern?) |  |  |  |  |  |
| … do you talk about topics like “displacement”, “refugees” and so on with your child?  (… sprechen Sie mit Ihrem Kind über Themen, wie “Flucht”, „Geflüchtete/ Flüchtlinge” usw.?) |  |  |  |  |  |

| **S3 Table. Original English version of adjectives, their German translation, and behavioural examples for the intergroup attitude task.** | | |
| --- | --- | --- |
| English [original] | German [translation] | Behavioural example [translated from German] |
| **Positive** | | |
| clean | sauber | Before children go to bed, they brush their teeth. |
| friendly | freundlich | Children help their parents carry the shopping. |
| good | artig | When children borrow a toy from another child, they hand it back. |
| hardworking | fleißig | Children help at home with baking and cooking. |
| kind | lieb | When a kitten is thirsty, children give it something to drink. |
| nice | nett | Children offer their toys to other children. |
| polite | höflich | When an elderly woman enters the train, children offer their seat. |
| **Negative** | | |
| bad | ungezogen | Children do things like drawing on the wall with crayons. |
| not nice | gemein | Children say nasty words to other people. |
| dirty | schmutzig | When children have dirty trousers, they put them on again. |
| unkind | nicht lieb | When children play with a doll, they are so rough that the doll breaks. |
| rude | unhöflich | Children do not share their birthday cake with other children. |
| lazy | faul | Children do not help to set the table at home. |
| unfriendly | unfreundlich | When children are in a bad mood, they do not talk to or play with other children. |
| *Note.* English adjectives based on Cameron et al. [2]. German version based on translation and back-translation to English. German versions of behavioural examples are available upon request. | | |

**Fig S1. Small drawings illustrating adjectives as part of the experimental interview set-up in the intergroup attitude task.**

*
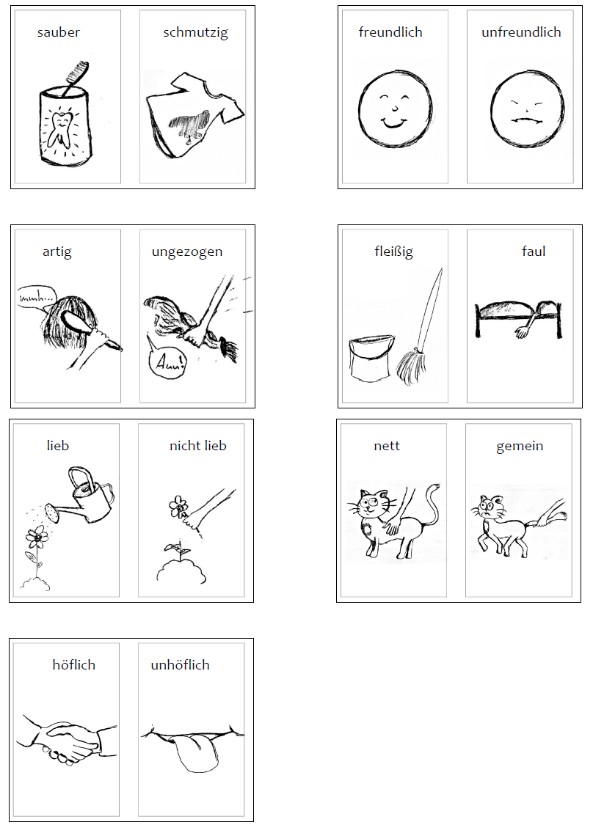
*

# Additional information on data analysis

| **S4 Table. Coding scheme of children’s drawings.** | | |
| --- | --- | --- |
| **No.** | **Category** | **Operationalization** |
| 1. | Size | Width X length (in cm^2^) |
| 2. | Number of colours in the drawing | Count of colours used in the drawing |
| 3. | Number of items in the drawing | Count of entities in the drawing, including persons, animals, and objects (two clouds = two entities) |
| 4. | Complexity of the figure | Count of body and facial parts (maximum of eight = 2 eyes, 1 nose, 1 mouth, 1 head, 2 arms, 2 legs) |
| 5. | Affect in the face of the depicted figure | Coder rating on 3-point-Likert-scale: 0-negative, 1-neutral, and 2-positive |
| 6. | Environment rating | Coder rating on 3-point-Likert-scale: 0-negative, 1-neutral, and 2-positive |
| 7. | Global similarity | Coder rating on 4-point-Likert-scale: 1-very much different, 2-rather different, 3-rather alike, 4-and very much alike. |
| *Note.* Categories coded per drawing, except for *7. Global similarity* (one code per participant). | | |

*Missing values*

We set the missing values for some of the count data — figure *size* and *complexity of the figure* (e.g., when a drawing included other entities but no target-child), and *number of items in the drawing* (e.g., when a drawing included the target-child but no other entities) — to ‘0’ to include the information (e.g. of a missing figure) into the results. There was also missing data for the ordinal *affect in the face of the depicted figure* (e.g., when the drawing included no human figure). Here we applied pairwise exclusion as interpreting a missing face as “negative”, “neutral” or “positive” would have been arbitrary. Pairwise exclusion applied to three cases: In one case, the participant drew neither a German nor a refugee child, and in two cases, participants drew a German but no refugee child.

For the intergroup attitude task, we calculated reliabilities of pre-scores by means of Cronbachs Alpha. Results are as follows: out-group positivity (α = 0.74), out-group negativity (α = 0.73), in-group negativity (α = 0.77), in-group positivity (α = 0.65).

*Test of normal distribution for all dependent variables*

For each dependent measure, we checked whether data was normally distributed, using Jarque-Bera tests [3]. Results indicate normally distributed data for the *intergroup attitude* and *group variability* in the intergroup attitude task, but not for any of the other variables (see S5 Table).

| **S5 Table. Outputs of the Jarque Bera Test results of normal distribution for all dependent variables.** | | | | |
| --- | --- | --- | --- | --- |
| Task | Variable | x-squared | df | p |
| Draw-a-typical-child task | Global similarity | 11.582 | 1 | 0.003 |
|  | Number of colours in the drawing | 8.647 | 2 | 0.013 |
|  | Number of items in the drawing | 308.89 | 2 | <0.001 |
|  | Complexity of the figure | 47.424 | 2 | <0.001 |
|  | Affect in the face of the figure | 82.237 | 2 | <0.001 |
|  | Environment rating | 4.503 | 2 | 0.105 |
|  | Size | 25.881 | 2 | <0.001 |
| Questions towards the drawings | Positivity towards the child in the picture | 7.956 | 2 | 0.019 |
| Intergroup attitude task | Intergroup attitude | 0.566 | 2 | 0.754 |
|  | Group variability | 3.516 | 2 | 0.172 |
| Liking task | Group liking | 11.177 | 2 | 0.004 |
| *Note.* Non-significant p-value > 0.05 indicates a normal distribution. | | | | |

| **S6 Table. Descriptive statistics (Means [M] and Standard Deviation [SD]) by contact opportunity and group for the draw-a-typical-child task.** | | | | | |  |
| --- | --- | --- | --- | --- | --- | --- |
|  |  | **Contact opportunity** | | **No contact opportunity** | | |
|  |  | ***M*** | ***(SD)*** | ***M*** | ***(SD)*** | |
| Size in cm | German child | 17.38 | (8.38) | 16.56 | (9.10) | |
|  | Refugee child | 17.37 | (8.01) | 15.44 | (9.95) | |
| Number of colours in the drawing | German child | 4.78 | (2.96) | 4.43 | (2.73) | |
|  | Refugee child | 4.50 | (2.86) | 4.25 | (2.22) | |
| Number of items in the drawing | German child | 2.75 | (4.72) | 1.64 | (2.91) | |
|  | Refugee child | 2.06 | (2.84) | 1.46 | (2.30) | |
| Complexity of the figure (no. of body and face parts) | German child | 9.09 | (1.55) | 9.14 | (2.82) | |
|  | Refugee child | 8.38 | (2.47) | 8.61 | (3.27) | |
| Figure has facial features (“yes”) | German child | 32/32 | | 27/28 | | |
|  | Refugee child | 30/32 | | 27/28 | | |
| Affect in the face of the depicted figure (3-point Likert scale)^A^ | German child | 2.70 | (0.60 | 2.85 | (0.36) | |
|  | Refugee child | 2.57 | (0.73) | 2.67 | (0.68) | |
| Environment rating (3-point Likert scale)^A^ | German child | 2.28 | (0.46) | 2.36 | (0.49) | |
|  | Refugee child | 2.28 | (0.52) | 2.18 | (0.55) | |
| *Note.* ^A^1-negative, 2-neutral, 3-positive | | | | | |  |

# Additional information on results

| **S7 Table. Frequentist ANOVA and Bayesian ANOVA results for categories coded in children’s drawings (draw-a-typical-child task).** | | | | |
| --- | --- | --- | --- | --- |
| Parameter | F(1, 57) | p | η^2^_g_ | BF_10_ |
| Size in cm |  |  |  |  |
| Contact^a^ | 0.13 | 0.718 | 0.002 | 0.44 ± 1.91% |
| Gender^b^ | 0.90 | 0.347 | 0.013 | 0.59 ± 0.68% |
| Group^c^ | 0.63 | 0.431 | 0.002 | 0.23 ± 1.92% |
| Contact x group | 0.82 | 0.369 | 0.002 | 0.30 ± 4.65% |
| Gender x group | 1.25 | 0.268 | 0.003 | 0.38 ± 2.88% |
| Number of colours in the drawing |  |  |  |  |
| Contact^a^ | 0.90 | 0.348 | 0.012 | 0.41 ± 2.20% |
| Gender^b^ | 28.44 | <0.001 | 0.286 | 7005.06 ± 1.16% |
| Group^c^ | 0.40 | 0.529 | 0.001 | 0.27 ± 1.29% |
| Contact x group | 0.32 | 0.571 | 0.001 | 0.27 ± 4.27% |
| Gender x group | 1.90 | 0.173 | 0.007 | 0.53 ± 2.45% |
| Number of items in the drawing |  |  |  |  |
| Contact^a^ | 0.39 | 0.533 | 0.006 | 0.56 ± 0.87% |
| Gender^b^ | 2.26 | 0.138 | 0.032 | 1.14 ± 3.43% |
| Group^c^ | 1.64 | 0.205 | 0.005 | 0.38 ± 0.78% |
| Contact x group | 0.23 | 0.635 | <0.001 | 0.33 ± 5.22% |
| Gender x group | 0.48 | 0.492 | 0.001 | 0.36 ± 6.62% |
| Complexity of the figure (no. of body and face parts) | | | | |
| Contact^a^ | 0.08 | 0.776 | 0.001 | 0.33 ± 0.59% |
| Gender^b^ | 0.04 | 0.834 | <0.001 | 0.33 ± 0.68% |
| Group^c^ | 3.58 | 0.063 | 0.013 | 1.22 ± 1.26% |
| Contact x group | 0.20 | 0.658 | <0.001 | 0.27 ± 2.35% |
| Gender x group | 0.35 | 0.559 | 0.001 | 0.30 ± 4.02% |
| Affect in the face of the depicted figure (3-point Likert scale)^A^ | | | | |
| Contact^a^ | 3.96 | 0.052 | 0.037 | 0.36 ± 1.82% |
| Gender^b^ | 8.75 | 0.005 | 0.077 | 1.81 ± 0.83% |
| Group^c^ | 1.35 | 0.251 | 0.012 | 0.59 ± 2.09% |
| Contact x group | 0.07 | 0.793 | <0.001 | 0.27 ± 2.90% |
| Gender x group | 2.50 | 0.120 | 0.022 | 1.07 ±15.01% |
| Environment rating (3-point Likert scale)^A^ | | | | |
| Contact^a^ | 0.56 | 0.459 | 0.008 | 0.33 ± 0.67% |
| Gender^b^ | 9.63 | 0.003 | 0.117 | 10.95 ± 1.60% |
| Group^c^ | 1.45 | 0.233 | 0.006 | 0.46 ± 1.00% |
| Contact x group | 0.83 | 0.367 | 0.003 | 0.66 ± 4.78% |
| Gender x group | 4.39 | 0.041 | 0.017 | 1.75 ±44.55% |
| *Note.* ^a^ Reference category ‘no direct contact’;  ^b^ Reference category ‘male’;  ^c^ Reference category ‘German child’.  ^A^ Pairwise exclusion for “Affect in the face of the depicted figure”. 1-negative, 2-neutral, 3-positive. | | | | |

1. Amt für Statistik Berlin-Brandenburg. Statistischer Bericht: Ergebnisse des Mikrozensus im Land Berlin 2018 - Bevölkerung und Erwerbstätigkeit 2019 [cited 26Aug.2020]. Available from: <https://www.statistik-berlin-brandenburg.de/publikationen/stat_berichte/2019/SB_A01-10-00_2018j01_BE.pdf>.

2. Cameron L, Rutland A, Brown R, Douch R. Changing Children’s Intergroup Attitudes towards Refugees: Testing Different Models of Extended Contact. Child Development. 2006;(77):1208-19. doi: 10.1111/j.1467-8624.2006.00929.x.

3. Hanusz Z, Enomoto R, Seo T, Koizumi K. A Monte Carlo comparison of Jarque–Bera type tests and Henze–Zirkler test of multivariate normality. Communications in Statistics - Simulation and Computation. 2018;47(5):1439-52. doi: 10.1080/03610918.2017.1315771.

# References
